# Donor *CYP3A5* Gene Polymorphism Alone Cannot Predict Tacrolimus Intrarenal Concentration in Renal Transplant Recipients

**DOI:** 10.3390/ijms21082976

**Published:** 2020-04-23

**Authors:** Mengyu Zhang, Soichiro Tajima, Tomohiro Shigematsu, Rao Fu, Hiroshi Noguchi, Keizo Kaku, Akihiro Tsuchimoto, Yasuhiro Okabe, Nobuaki Egashira, Satohiro Masuda

**Affiliations:** 1 Department of Clinical Pharmacology and Biopharmaceutics, Graduate School of Pharmaceutical Sciences, Kyushu University, 3-1-1 Maidashi, Higashi-ku, Fukuoka 812-8582, Japan; zhang.mengyu.347@s.kyushu-u.ac.jp (M.Z.); shige825@pharm.med.kyushu-u.ac.jp (T.S.); fu.rao.500@s.kyushu-u.ac.jp (R.F.); n-egashi@pharm.med.kyushu-u.ac.jp (N.E.); 2 Department of Pharmacy, Kyushu University Hospital, 3-1-1 Maidashi, Higashi-ku, Fukuoka 812-8582, Japan; stajima@pharm.med.kyushu-u.ac.jp; 3 Department of Surgery and Oncology, Graduate School of Medical Sciences, Kyushu University, 3-1-1 Maidashi, Higashi-ku, Fukuoka 812-8582, Japan; noguchih@med.kyushu-u.ac.jp (H.N.); kaku@med.kyushu-u.ac.jp (K.K.); yokabe1970@gmail.com (Y.O.); 4 Department of Medicine and Clinical Science, Graduate School of Medical Sciences, Kyushu University, 3-1-1 Maidashi, Higashi-ku, Fukuoka 812-8582, Japan; tucimoto@intmed2.med.kyushu-u.ac.jp; 5 Department of Pharmacy, International University of Health and Welfare Narita Hospital, 852 Hatakeda, Narita 286-0124, Japan; 6 Department of Clinical Pharmacy, Faculty of Pharmaceutical Sciences, International University of Health and Welfare, 2600-1 Kita-kanemaru, Otawara 324-8501, Japan

**Keywords:** tacrolimus, renal transplantation, intrarenal concentration, whole blood concentration, LC-MS/MS

## Abstract

*CYP3A5* gene polymorphism in recipients plays an important role in tacrolimus blood pharmacokinetics after renal transplantation. Even though CYP3A5 protein is expressed in renal tubular cells, little is known about the influence on the tacrolimus intrarenal exposure and hence graft outcome. The aim of our study was to investigate how the tacrolimus intrarenal concentration (C_tissue_) could be predicted based on donor *CYP3A5* gene polymorphism in renal transplant recipients. A total of 52 Japanese renal transplant patients receiving tacrolimus were enrolled in this study. Seventy-four renal biopsy specimens were obtained at 3 months and 1 year after transplantation to determine the donor *CYP3A5* polymorphism and measure the C_tissue_ by liquid chromatography-tandem mass spectrometry (LC-MS-MS). The tacrolimus C_tissue_ ranged from 52 to 399 pg/mg tissue (*n* = 74) and was weak but significantly correlated with tacrolimus trough concentration (C_0_) at 3 months after transplantation (Spearman, *r* = 0.3560, *p* = 0.0096). No significant relationship was observed between the donor *CYP3A5* gene polymorphism and C_tissue_ or C_tissue_/C_0_. These data showed that the tacrolimus systemic level has an impact on tacrolimus renal accumulation after renal transplantation. However, donor *CYP3A5* gene polymorphism alone cannot be used to predict tacrolimus intrarenal exposure_._ This study may be valuable for exploring tacrolimus renal metabolism and toxicology mechanism in renal transplant recipients.

## 1. Introduction

**Figure 1:**
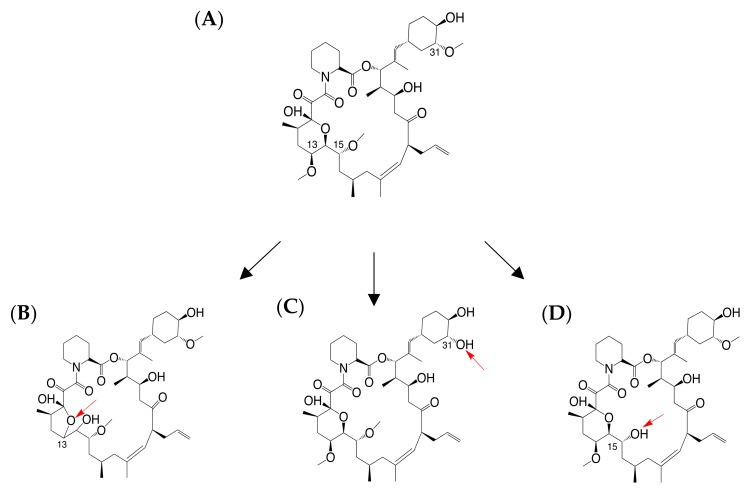
Chemical structure of (**A**) tacrolimus; (**B**) 13-O-desmethyl tacrolimus (M1); (**C**) 31-O-desmethyl tacrolimus (M2); (**D**) 15-O-desmethyl tacrolimus (M3). Each site of metabolism is indicated by a red arrow in the corresponding metabolite structures, respectively.

Tacrolimus is a commonly used calcineurin inhibitor (CNI) for immunosuppressive therapy to prevent allograft rejection after renal transplantation. With a narrow therapeutic index and high inter-patient pharmacokinetic variability, monitoring the tacrolimus trough concentration (C_0_) in whole blood has been recommended in post-transplantation clinical routine to optimize the therapy outcome and minimize adverse effects [1,2]. However, within target therapeutic range, the risk of adverse events such as acute rejection (AR) could not be completely avoided [3]. Tacrolimus is predominantly metabolized by hepatic and intestinal cytochrome P450 (CYP) enzymes. Three desmethyl metabolites, namely, 13-O-desmethyl tacrolimus (M1), 31-O-desmethyl tacrolimus (M2), and 15-O-desmethyl tacrolimus (M3), have been identified as the major tacrolimus metabolites [4] (Figure 1) and might contribute to the tacrolimus-induced toxic effects [5,6,7].

The loss-of-function *CYP3A5* 6986A>G (CYP3A5*3) polymorphism significantly affects the metabolism and pharmacokinetics of tacrolimus [8,9]. Patients with CYP3A5*1 allele require a higher tacrolimus dose to reach target C_0_ levels than those with CYP3A5*3/*3 genotype [10,11,12]. Even though tacrolimus is primarily metabolized by CYP3A5 protein in the liver and small intestine, it has been reported that the CYP3A5 protein is also expressed in renal tubular epithelial cells [13]. The human kidney microsomes with CYP3A5*1 allele was associated with a higher metabolic activity compared to those with CYP3A5*3/*3 genotype [14]. Therefore, some studies have hypothesized that tacrolimus intrarenal concentration (C_tissue_) could be affected by the graft *CYP3A5* gene polymorphism (donor genotype) and suggested that the tacrolimus intrarenal levels could be a better predictor of histological AR than blood concentrations [10,15]. However, so far, there have been only two studies that have preliminarily measured the tacrolimus concentration in human kidney tissues [16,17], and little clinical information is available about association between the donor *CYP3A5* gene polymorphism and tacrolimus renal metabolism. Therefore, in this study, we investigated the potential factors (tacrolimus blood levels and donor *CYP3A5* gene polymorphism) that determine tacrolimus intrarenal exposure and the relationship between tacrolimus intrarenal level and biopsy-proven subclinical AR (subAR) in renal transplant recipients. We focused on evaluating the capability of utilizing donor *CYP3A5* gene polymorphism to predict tacrolimus C_tissue_ in renal transplant recipients.

## 2. Results

### 2.1. Patient Characteristics and CYP3A5 Polymorphism

All patients included in this study underwent at least one protocol renal biopsy (*n* = 52). A total of 74 renal biopsies were obtained: 52 biopsies and 22 biopsies at 3 months and 1 year after transplantation, respectively. The demographic data of recipients admitted in this study are shown in Table 1. The mean recipient age was observed to be 43.9 ± 13.3 years and their mean body weight was 58.15 ± 14.48 kg. The frequencies for *CYP3A5* genotypes in both donors and recipients are summarized in Table 1. Among the 52 renal transplant recipients and their corresponding donors, 23 (44.2%) recipients and 25 (48.1%) donors exhibited *1/*1 or *1/*3 genotype, while 29 (55.8%) recipients and 27 (51.9%) donors carried *3/*3 genotype. The allele frequencies for CYP3A5*3 in donors and recipients were 71.2% and 74.0%, respectively. The results were consistent with minor allele frequency of CYP3A5*3 in Asian population as reported in [18].

### 2.2. Influence of CYP3A5 Polymorphism on Tacrolimus Pharmacokinetics

We evaluated the influence of the donor and recipient *CYP3A5* polymorphism on tacrolimus pharmacokinetics by assessing C_0_ and dose-adjusted C_0_ (C_0_/D) of tacrolimus. No significant relationship was observed between the donor *CYP3A5* polymorphism and tacrolimus pharmacokinetics. On the other hand, CYP3A5*3 allele in recipients had a significant impact on C_0_/D as reported by previous studies [19,20,21]. Recipients with CYP3A5*3/*3 had a significantly higher C_0_/D than those with CYP3A5*3 allele at both 3 months and 1 year after renal transplantation (*p* < 0.0001 and *p* = 0.0167, respectively) (Table 2).

### 2.3. Association between Intrarenal and Whole Blood Tacrolimus Levels

**Figure 2:**
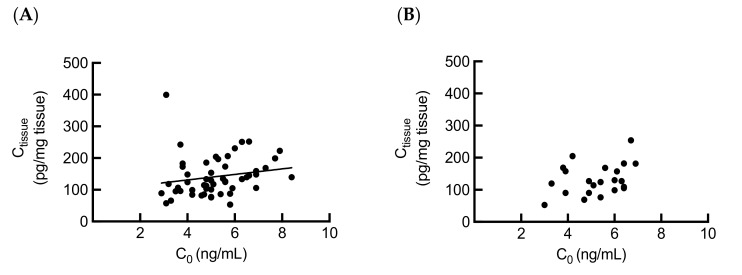
Correlation between the tacrolimus C_0_ and C_tissue_ at (**A**) 3 months (*n* = 52, *r* = 0.3560, *p* = 0.0096) and (**B**) 1 year (*n* = 22, *r* = 0.3368, *p* = 0.1253) after transplantation. Statistical analyses were performed using Spearman’s correlation.

Since a previous study has reported that in renal transplant recipients, changes in tacrolimus intrarenal concentrations reflect the blood concentration of tacrolimus [17], we investigated the association between the tacrolimus C_0_ and C_tissue_ at 3 months and 1 year after renal transplantation, respectively. A correlation (*r* = 0.3560, *p* = 0.0096) between the tacrolimus C_tissue_ and C_0_ was observed only at 3 months after transplantation (Figure 2). This result implies that the tacrolimus blood levels may affect tacrolimus intrarenal accumulation.

### 2.4. Influence of Donor CYP3A5 Gene Polymorphism on Tacrolimus Metabolism in Kidney

**Figure 3:**
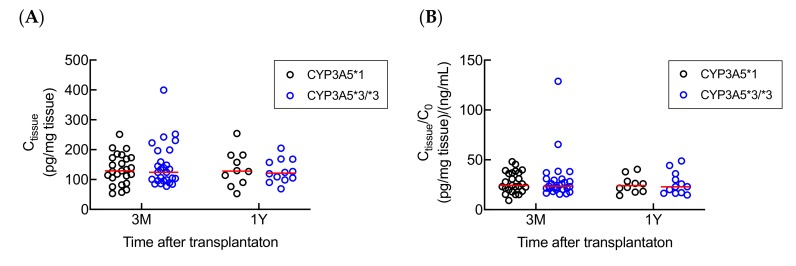
Effects of donor-*CYP3A5* genotype on tacrolimus (**A**) C_tissue_ (3 months: *p* = 0.8845; 1 year: *p* = 0.6873) and (**B**) C_tissue_/C_0_ (3 months: *p* = 0.7575; 1 year: *p* = 0.9229) at 3 months (*n* = 52) and 1 year (*n* = 22) after transplantation. Statistical analyses were performed using Mann–Whitney U test. Bar shows the median value in each group. (Abbreviations: 3M—3 months; 1Y—1 year).

**Figure 4:**
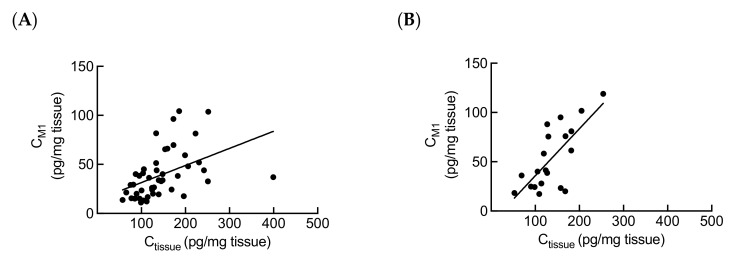
Correlation between the intrarenal concentrations of tacrolimus and M1 at (**A**) 3 months (*n* = 52, *r* = 0.6008, *p* < 0.0001) and (**B**) 1 year (*n* = 22, *r* = 0.6632, *p* = 0.0014) after transplantation. Statistical analyses were performed using Spearman’s correlation.

*CYP3A5* polymorphism in kidney has been reported to influence local metabolism of tacrolimus in vitro [14]. Therefore, to verify the role of *CYP3A5* polymorphism in tacrolimus renal metabolism in vivo, the association between the donor *CYP3A5* polymorphism and tacrolimus C_tissue_ or C_tissue_/C_0_ was estimated. No significant relationship was observed between tacrolimus C_tissue_ or C_tissue_/C_0_ and donor *CYP3A5* polymorphism at both 3 months and 1 year after transplantation (Figure 3). To further investigate the metabolism of tacrolimus in kidney, we measured the intrarenal concentrations of three major tacrolimus metabolites (M1, M2, and M3) in 74 biopsy samples, and of these 74 samples, 66 (89.2%), 15 (20.3%), and 3 (4.1%) samples had M1, M2, and M3 concentrations above the lower limit of quantification (LLOQ, 0.01 ng/mL), respectively. We found that the mean intrarenal concentrations of M1, M2, and M3 were 29.1%, 8.43%, and 5.18% of the tacrolimus intrarenal concentrations, respectively (data not shown). We also observed that M1 intrarenal concentration (C_M1_) was significantly associated with the tacrolimus C_tissue_ in patients both at 3 months and 1 year after transplantation (Figure 4).

### 2.5. Associations between Subclinical Acute Rejection (subAR) and Tacrolimus C_tissue_ or C_tissue_/C_0_.

**Figure 5:**
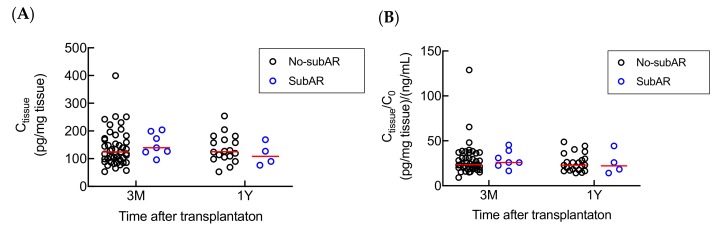
Differences in tacrolimus (**A**) C_tissue_ (3 months: *p* = 0.3287; 1 year: *p* = 0.5468) and (**B**) C_tissue_/C_0_ (3 months: *p* = 0.3699, 1 year: *p* = 0.7743) among the no subclinical acute rejection (no-subAR) group and subAR group at 3 months (*n* = 52) and 1 year (*n* = 22) after transplantation respectively. Statistical analyses were performed using Mann–Whitney U test. Bar shows the median value in each group. (Abbreviations: 3M—3 months; 1Y—1 year).

A total of 7 (13.5%) and 4 (18.2%) patients were diagnosed with biopsy-proven subclinical acute rejection (subAR) at 3 months and 1 year after renal transplantation, respectively. By comparing the tacrolimus C_tissue_ and C_tissue_/C_0_ between no-subAR and subAR group of patients, no significant difference was found either at 3 months or 1 year after renal transplantation (Figure 5).

## 3. Discussion

The pharmacogenetics studies of tacrolimus have focused on the impact of recipient *CYP3A5* genotype on the tacrolimus pharmacokinetics; however, the role of donor *CYP3A5* polymorphism in tacrolimus systemic or local metabolism is little to know. In this study, we investigated the influence of donor *CYP3A5* polymorphism on tacrolimus pharmacokinetics at 3 months and 1 year after renal transplantation. There was no effect of donor *CYP3A5* polymorphism on the tacrolimus C_0_ or C_0_/D, as predicted. As the CYP3A5 metabolic capability in the kidney is not comparable to that in liver and intestine of recipients, the donor *CYP3A5* polymorphism is unlikely to contribute significantly to the systemic levels of tacrolimus [14]. Next, we investigated the relationship between the tacrolimus C_0_ and C_tissue_. Interestingly, we found a significant correlation between the tacrolimus C_0_ and C_tissue_ at 3 months after transplantation. This result was contrary to that in the study by Noll et al. [17], and this could be due to the differences in post-transplantation stage and sample size.

Previous reports have shown that the intrarenal concentration of tacrolimus or its metabolites could be affected by *CYP3A5* genotype, and further predict the clinical outcome and side effects in immunosuppressive therapy [14,15,22]. On the other hand, it has been reported that renal *CYP3A5* polymorphism may influence the development of tacrolimus-related nephrotoxicity in liver transplant patients [23]. In the present study, the donor (graft kidney) *CYP3A5* polymorphism was examined as to whether it was effective on tacrolimus local metabolism in renal transplant recipients. However, we found no significant relationship between the donor CYP3A5*3 polymorphism and C_tissue_ or C_tissue_/C_0_ after transplantation. There are several possible reasons for this result. Firstly, the expression levels and protein distribution of CYP3A5 could be variable in different areas of the kidney. Despite the fact that CYP3A5*1 allele was shown to be associated with a higher CYP3A5 expression compared to CYP3A5*3/*3, the expression difference reported was limited to the proximal tubule [24]. In the present study, we were unable to ensure that all biopsies were sampled from the same position of kidney, and the data of CYP3A5 expression levels and tacrolimus intrarenal distribution were not available. Secondly, we could not exclude the possibility that ischemia reperfusion injury occurred after renal transplantation, which might have had an impact on the expression levels of CYP3A5 in the allograft organ [25,26]. Moreover, it is likely that the effect of donor *CYP3A5* polymorphism on local renal metabolism could be counterbalanced by the influence of tacrolimus systemic concentration. This result is consistent with the findings of Kuypers et al. [5]. They demonstrated that the recipient CYP3A5*1 variant is associated with tacrolimus-related nephrotoxicity and suggested that this is possibly due to higher concentrations of toxic metabolites in kidney tissue. Consequently, recipient (liver and intestine) *CYP3A5* polymorphism plays an important role in the renal accumulation of tacrolimus compared to donor (graft kidney) *CYP3A5* polymorphism.

In addition to CYP3A5, other genetic and clinical factors could be potential biomarkers to predict the tacrolimus C_tissue_. Especially, the drug efflux transporter *ABCB1* is also closely related to the metabolism of tacrolimus. The activity and expression levels of *ABCB1* could contribute to the variability in tacrolimus absorption and excretion [23,27,28]. Even though *ABCB1* polymorphisms’ impact on tacrolimus pharmacokinetics is still unclear [10,29,30,31], the *ABCB1* polymorphism has been identified as a critical factor in intracellular tacrolimus exposure [32,33]. It is widely accepted that *ABCB1* polymorphism and expression levels are more likely be associated with tacrolimus tissue distribution and drug effect or toxicity in the allograft [18,34,35,36]. Moreover, Knops et al. demonstrated that the *ABCB1* 3435T variant in human proximal tubule cells has a significant impact on tacrolimus metabolism in vitro [37]. Moreover, *ABCB1*, but not *CYP3A5*, polymorphisms in the liver have been reported to significantly influence tacrolimus hepatic concentrations in liver transplant recipients [38]. Therefore, it is reasonable to expect that *ABCB1* polymorphisms might contribute to the regulation of tacrolimus intrarenal metabolism.

In this study, we also measured simultaneously the concentrations of three major tacrolimus metabolites M1, M2, and M3 (Figure 1) in kidney tissues. We found M1 as the most abundant metabolite of tacrolimus in kidney, as is the case with human liver microsomes and blood reported by previous studies [39,40,41,42]. As M1 retains around 10% of tacrolimus immunosuppressant activity [4], the clinical value and potential toxic property of M1 intrarenal exposure need to be further studied. Although we could not detect the three metabolites in all biopsy samples in this study, these results may be useful for explaining the mechanism of tacrolimus-induced nephrotoxicity.

There is an ongoing debate on whether tacrolimus C_0_ could accurately predict or reflect acute rejection. Alternate matrices such as intracellular or allograft tissue, are recommended to improve tacrolimus treatment monitoring in recent years [43]. In fact, we indeed observed several patients who showed relatively low tacrolimus C_tissue_ despite their tacrolimus C_0_ in the normal therapeutic range. Nevertheless, we found no significant association between the tacrolimus C_tissue_ or C_tissue_/C_0_ and the incidence of biopsy-proven subAR in our study. However, since the sample size was relatively small, whether the tacrolimus intrarenal levels could better reflect the incidence of AR or subAR than C_0_ requires further investigation. Moreover, new biomarkers predictive of tacrolimus intrarenal level would be explored in future mechanistic and clinical monitoring studies to identify patients who are at high risk of adverse events but with a target tacrolimus therapeutic concentration in blood.

## 4. Materials and Methods

### 4.1. Subjects

A total of 59 Japanese adult renal transplant patients were enrolled in this study. All patients underwent renal transplantation between August 2014 and August 2016 at Kyushu University Hospital in Japan. The study excluded 7 patients who received everolimus in immunosuppressive therapy after transplantation. Ultimately, our study comprised of 52 renal transplant recipients. All 52 recipients received a triple-drug regimen comprising of tacrolimus, mycophenolate mofetil, and prednisolone. This study was conducted in accordance with the Declaration of Helsinki and its amendments and was approved by the Institutional Review Board of Kyushu University Graduate School and Faculty of Medicine (approval number: 588-00, 28 July 2014). All patients enrolled in this study gave written informed consent for participation in the study and for the use of their sample.

### 4.2. Diagnostic Criteria for Subclinical Acute Rejection (SubAR)

Protocol biopsies were performed 3 months and 1 year after kidney transplantation. Each biopsy was graded according to Banff 2009 classification and diagnosed for subclinical acute rejection (SubAR). SubAR was identified by the presence of tubulointerstitial mononuclear infiltration with a requirement of <10% rise in serum creatinine in 2 weeks before the protocol biopsy and no absence of clinical functional deterioration [44].

### 4.3. Measurement of Tacrolimus Trough Concentration

To determine tacrolimus C_0,_ whole venous blood was obtained from patients before they received the morning dose of tacrolimus. Tacrolimus C_0_ was measured using a Chemiluminescent Immunoassay (Architect; Abbott Park, Illinois, USA).

### 4.4. Measurement of Tacrolimus Renal Concentration

A fraction of kidney biopsies (1–3 mg of wet tissue) was used for intrarenal tacrolimus quantification, which was performed on a Shimadzu LCMS-8050 liquid triple quadrupole tandem mass spectrometer (Shimadzu, Kyoto, Japan). Samples were prepared using a procedure described by Krogstad et al. with slight modification [16]. The frozen renal biopsy was dried on filter paper for 90 min at room temperature. Once dry, the biopsy was weighed and homogenized in 100 μL ultrapure water by syringe and needle. Aliquot 50 μL of tissue homogenate was transferred to a 1.5 mL microcentrifuge tube, and 20 μL methanol was added then vortexed for 30 s. Then, aliquot 80 μL of protein precipitation solution (1 ng/mL ascomycin as internal standard in 70/30 methanol/zinc sulphate 0.1 mol/L) was added to the tube, then vortexed at 1500 rpm for 15 min. After centrifugation at 9400× *g* for 10 min, the supernatant was transferred to vials and injected to a LC-MS system. Quantitation was performed with a GL Sciences Inertsil-ODS-3 (3 μm; 2.1 mm × 150 mm) column. Mobile phase A consisted of 2 mmol/L ammonium acetate with 0.1% formic acid (*v/v*) in water, and mobile phase B consisted of 2 mmol/L ammonium acetate with 0.1% formic acid (*v/v*) in methanol. The gradient was started at 60% B, increased to 85% B at 3 min, increased to 95% B at 6 min, increased to 100% B at 6.5 min, switched back to the starting conditions at 60% B from 6.5 min to 6.6 min, and re-equilibration for 1.4 min. The total analysis time was 8 min. The flow rate was 0.25 mL/min, and the column temperature was maintained at 60 °C. Electrospray ionization was performed in a positive mode. Analysis was based on multiple reaction monitoring (MRM) of m/z 821.40→768.35 for tacrolimus, 807.20→754.25 for M1/M2/M3, and m/z 809.3→756.3 for internal standard. The calibration curve ranges were 0.02–2 ng/mL and 0.01–1 ng/mL for tacrolimus and tacrolimus metabolites, respectively.

### 4.5. Statistical Analysis

Statistical analysis was performed using Prism 8.0 (GraphPad Software, Inc., San Diego, CA, USA). Mann–Whitney U test was used to compare the differences between tacrolimus concentrations in *CYP3A5* genotype groups and in patients with/without subAR. Correlations between tacrolimus C_tissue_ and C_0_ or M1 intrarenal concentration were analyzed by Spearman’s correlation. A *p*-value < 0.05 was considered to be statistically significant.

## 5. Conclusions

This study, for the first time, demonstrated a correlation between tacrolimus C_tissue_ and C_0_ and that donor *CYP3A5* gene polymorphism alone was not sufficient to predict the renal concentration of tacrolimus at 3 months and 1 year after renal transplantation. Future studies will need to investigate the intrarenal concentration of tacrolimus or its metabolites and the adverse events in a large clinical study.

## Figures and Tables

**Table 1:** Characteristics of patients.

Characteristics	*n* = 52
Recipient age (years)	43.9 ± 13.3
Recipient sex (male/female)	31/21
Body weight (kg)	58.15 ± 14.48
Reasons for renal transplantation (n)	
IgA nephropathy	8
Diabetic gastropathy	8
Chronic glomerulonephritis	10
Polycystic kidney	3
Type 1 diabetes	2
Type 2 diabetes	3
Hypertensive nephrosclerosis	3
Others	15
Serum creatinine (mg/dL)	
Pre-transplant	7.85 ± 3.38
3-month	1.14 ± 0.28
Donor *CYP3A5* genotype	
*1/*1 or *1/*3	25 (48.1%)
*3/*3	27 (51.9%)
Recipient *CYP3A5* genotype	
*1/*1 or *1/*3	23 (44.2%)
*3/*3	29 (55.8%)

Data are expressed as mean ± standard deviation, number (%).

**Table 2:** Tacrolimus pharmacokinetics (PK) parameter according to *CYP3A5* genotype.

PK-Parameter	*CYP3A5* Genotype	3 Months after Renal Transplantation (*n* = 52)			1 Year after Renal Transplantation (*n* = 22)		
		*n*	Mean ± SD	*p*	*n*	Mean ± SD	*p*
**C_0_ (ng/mL)**	Recipient CYP3A5*1	23	5.30 ± 1.32	0.5368	8	5.01 ± 1.45	0.7002
	Recipient CYP3A5*3/*3	29	5.08 ± 1.35		14	5.37 ± 1.00	
C_0_/D (ng/mL/mg)	Recipient CYP3A5*1	23	0.95 ± 0.37	<0.0001	8	0.97 ± 0.37	0.0167
	Recipient CYP3A5*3/*3	29	1.56 ± 0.66		14	1.58 ± 0.68	
C_0_ (ng/mL)	Donor CYP3A5*1	25	5.03 ± 1.11	0.5760	10	5.32 ± 1.24	0.7339
	Donor CYP3A5*3/*3	27	5.31 ± 1.51		12	5.18 ± 1.14	
C_0_/D (ng/mL/mg)	Donor CYP3A5*1	25	1.31 ± 0.69	0.9964	10	1.43 ± 0.60	0.4078
	Donor CYP3A5*3/*3	27	1.27 ± 0.57		12	1.30 ± 0.71	

## Abbreviations

C_0_	Tacrolimus trough concentration
C_tissue_	Tacrolimus intrarenal concentration
CNI	Calcineurin inhibitor
AR	Acute rejection
SubAR	Subclinical acute rejection
C_0_/D	Dose-adjusted C_0_
C_M1_	Intrarenal concentration of 13-O-desmethyl tacrolimus

## References

[1] De Jonge H., Naesens M., Kuypers D.R. (2009). New insights into the pharmacokinetics and pharmacodynamics of the calcineurin inhibitors and mycophenolic acid: Possible consequences for therapeutic drug monitoring in solid organ transplantation. Ther. Drug Monit..

[2] Schiff J., Cole E., Cantarovich M. (2007). Therapeutic monitoring of calcineurin inhibitors for the nephrologist. Clin. J. Am. Soc. Nephrol..

[3] Bouamar R., Shuker N., Hesselink D.A., Weimar W., Ekberg H., Kaplan B., Bernasconi C., Van Gelder T. (2013). Tacrolimus predose concentrations do not predict the risk of acute rejection after renal transplantation: A pooled analysis from three randomized-controlled clinical trials(†). Am. J. Transplant..

[4] Iwasaki K., Shiraga T., Nagase K., Tozuka Z., Noda K., Sakuma S., Fujitsu T., Shimatani K., Sato A., Fujioka M. (1993). Isolation, identification, and biological activities of oxidative metabolites of FK506, a potent immunosuppressive macrolide lactone. Drug Metab. Dispos..

[5] Kuypers D.R., De Jonge H., Naesens M., Lerut E., Verbeke K., Vanrenterghem Y. (2007). CYP3A5 and CYP3A4 but not MDR1 single-nucleotide polymorphisms determine long-term tacrolimus disposition and drug-related nephrotoxicity in renal recipients. Clin. Pharm..

[6] Zegarska J., Hryniewiecka E., Zochowska D., Samborowska E., Jazwiec R., Borowiec A., Tszyrsznic W., Chmura A., Nazarewski S., Dadlez M. (2016). Tacrolimus Metabolite M-III May Have Nephrotoxic and Myelotoxic Effects and Increase the Incidence of Infections in Kidney Transplant Recipients. Transplant. Proc..

[7] Zegarska J., Hryniewiecka E., Zochowska D., Samborowska E., Jazwiec R., Maciej K., Nazarewski S., Dadlez M., Paczek L. (2018). Evaluation of the Relationship Between Concentrations of Tacrolimus Metabolites, 13-O-Demethyl Tacrolimus and 15-O-Demethyl Tacrolimus, and Clinical and Biochemical Parameters in Kidney Transplant Recipients. Transplant. Proc..

[8] Hesselink D.A., Van Schaik R.H., Van der Heiden I.P., Van der Werf M., Gregoor P.J., Lindemans J., Weimar W., Van Gelder T. (2003). Genetic polymorphisms of the CYP3A4, CYP3A5, and MDR-1 genes and pharmacokinetics of the calcineurin inhibitors cyclosporine and tacrolimus. Clin. Pharm..

[9] Chen L., Prasad G.V.R. (2018). CYP3A5 polymorphisms in renal transplant recipients: Influence on tacrolimus treatment. Pharmgenomics Pers. Med..

[10] Glowacki F., Lionet A., Buob D., Labalette M., Allorge D., Provôt F., Hazzan M., Noël C., Broly F., Cauffiez C. (2011). CYP3A5 and ABCB1 polymorphisms in donor and recipient: Impact on Tacrolimus dose requirements and clinical outcome after renal transplantation. Nephrol. Dial. Transplant..

[11] Wang L., Liu L.H., Tong W.H., Wang M.X., Lu S.C. (2015). Effect of CYP3A5 gene polymorphisms on tacrolimus concentration/dosage ratio in adult liver transplant patients. Genet. Mol. Res..

[12] Zhang X., Liu Z.H., Zheng J.M., Chen Z.H., Tang Z., Chen J.S., Li L.S. (2005). Influence of CYP3A5 and MDR1 polymorphisms on tacrolimus concentration in the early stage after renal transplantation. Clin. Transplant..

[13] Haehner B.D., Gorski J.C., Vandenbranden M., Wrighton S.A., Janardan S.K., Watkins P.B., Hall S.D. (1996). Bimodal distribution of renal cytochrome P450 3A activity in humans. Mol. Pharm..

[14] Dai Y., Hebert M.F., Isoherranen N., Davis C.L., Marsh C., Shen D.D., Thummel K.E. (2006). Effect of CYP3A5 polymorphism on tacrolimus metabolic clearance in vitro. Drug Metab. Dispos..

[15] Zheng S., Tasnif Y., Hebert M.F., Davis C.L., Shitara Y., Calamia J.C., Lin Y.S., Shen D.D., Thummel K.E. (2012). Measurement and compartmental modeling of the effect of CYP3A5 gene variation on systemic and intrarenal tacrolimus disposition. Clin. Pharm..

[16] Krogstad V., Vethe N.T., Robertsen I., Hasvold G., Ose A.D., Hermann M., Andersen A.M., Chan J., Skauby M., Svensson M.H.S. (2018). Determination of Tacrolimus Concentration and Protein Expression of P-Glycoprotein in Single Human Renal Core Biopsies. Drug Monit..

[17] Noll B.D., Coller J.K., Somogyi A.A., Morris R.G., Russ G.R., Hesselink D.A., Van Gelder T., Sallustio B.C. (2013). Validation of an LC-MS/MS method to measure tacrolimus in rat kidney and liver tissue and its application to human kidney biopsies. Drug Monit..

[18] Brunet M., Van Gelder T., Åsberg A., Haufroid V., Hesselink D.A., Langman L., Lemaitre F., Marquet P., Seger C., Shipkova M. (2019). Therapeutic Drug Monitoring of Tacrolimus-Personalized Therapy: Second Consensus Report. Drug Monit..

[19] Chen J.S., Li L.S., Cheng D.R., Ji S.M., Sun Q.Q., Cheng Z., Wen J.Q., Sha G.Z., Liu Z.H. (2009). Effect of CYP3A5 genotype on renal allograft recipients treated with tacrolimus. Transplant. Proc..

[20] Goto M., Masuda S., Kiuchi T., Ogura Y., Oike F., Okuda M., Tanaka K., Inui K. (2004). CYP3A5*1-carrying graft liver reduces the concentration/oral dose ratio of tacrolimus in recipients of living-donor liver transplantation. Pharmacogenetics.

[21] Kato H., Usui M., Muraki Y., Tanemura A., Murata Y., Kuriyama N., Azumi Y., Kishiwada M., Mizuno S., Sakurai H. (2016). Long-Term Influence of CYP3A5 Gene Polymorphism on Pharmacokinetics of Tacrolimus and Patient Outcome After Living Donor Liver Transplantation. Transplant. Proc..

[22] Vanhove T., De Jonge H., De Loor H., Oorts M., De Hoon J., Pohanka A., Annaert P., Kuypers D.R.J. (2018). Relationship between In Vivo CYP3A4 Activity, CYP3A5 Genotype, and Systemic Tacrolimus Metabolite/Parent Drug Ratio in Renal Transplant Recipients and Healthy Volunteers. Drug Metab. Dispos..

[23] Fukudo M., Yano I., Yoshimura A., Masuda S., Uesugi M., Hosohata K., Katsura T., Ogura Y., Oike F., Takada Y. (2008). Impact of MDR1 and CYP3A5 on the oral clearance of tacrolimus and tacrolimus-related renal dysfunction in adult living-donor liver transplant patients. Pharm. Genom..

[24] Bolbrinker J., Seeberg S., Schostak M., Kempkensteffen C., Baelde H., De Heer E., Kreutz R. (2012). CYP3A5 genotype-phenotype analysis in the human kidney reveals a strong site-specific expression of CYP3A5 in the proximal tubule in carriers of the CYP3A5*1 allele. Drug Metab. Dispos..

[25] Knops N., Levtchenko E., Van den Heuvel B., Kuypers D. (2013). From gut to kidney: Transporting and metabolizing calcineurin-inhibitors in solid organ transplantation. Int. J. Pharm..

[26] Wang B.Y., Li Q.X., Li J., Xie X.F., Ao Y., Ai Y.X. (2009). Hepatotoxicity and gene expression down-regulation of CYP isozymes caused by renal ischemia/reperfusion in the rat. Exp. Toxicol. Pathol..

[27] Fu R., Tajima S., Suetsugu K., Watanabe H., Egashira N., Masuda S. (2019). Biomarkers for individualized dosage adjustments in immunosuppressive therapy using calcineurin inhibitors after organ transplantation. Acta Pharm. Sin..

[28] Kravljaca M., Perovic V., Pravica V., Brkovic V., Milinkovic M., Lausevic M., Naumovic R. (2016). The importance of MDR1 gene polymorphisms for tacrolimus dosage. Eur. J. Pharm. Sci..

[29] Staatz C.E., Goodman L.K., Tett S.E. (2010). Effect of CYP3A and ABCB1 single nucleotide polymorphisms on the pharmacokinetics and pharmacodynamics of calcineurin inhibitors: Part II. Clin. Pharmacokinet..

[30] Tsuchiya N., Satoh S., Tada H., Li Z., Ohyama C., Sato K., Suzuki T., Habuchi T., Kato T. (2004). Influence of CYP3A5 and MDR1 (ABCB1) polymorphisms on the pharmacokinetics of tacrolimus in renal transplant recipients. Transplantation.

[31] Stefanović N.Z., Cvetković T.P., Jevtović-Stoimenov T.M., Ignjatović A.M., Paunović G.J., Veličković R.M. (2015). Investigation of CYP 3A5 and ABCB1 gene polymorphisms in the long-term following renal transplantation: Effects on tacrolimus exposure and kidney function. Exp. Med..

[32] Capron A., Mourad M., De Meyer M., De Pauw L., Eddour D.C., Latinne D., Elens L., Haufroid V., Wallemacq P. (2010). CYP3A5 and ABCB1 polymorphisms influence tacrolimus concentrations in peripheral blood mononuclear cells after renal transplantation. Pharmacogenomics.

[33] Dessilly G., Elens L., Panin N., Capron A., Decottignies A., Demoulin J.B., Haufroid V. (2014). ABCB1 1199G>A genetic polymorphism (Rs2229109) influences the intracellular accumulation of tacrolimus in HEK293 and K562 recombinant cell lines. PLoS ONE.

[34] Bandur S., Petrasek J., Hribova P., Novotna E., Brabcova I., Viklicky O. (2008). Haplotypic structure of ABCB1/MDR1 gene modifies the risk of the acute allograft rejection in renal transplant recipients. Transplantation.

[35] Naesens M., Lerut E., De Jonge H., Van Damme B., Vanrenterghem Y., Kuypers D.R. (2009). Donor age and renal P-glycoprotein expression associate with chronic histological damage in renal allografts. J. Am. Soc. Nephrol..

[36] Yigitaslan S., Erol K., Cengelli C. (2016). The Effect of P-Glycoprotein Inhibition and Activation on the Absorption and Serum Levels of Cyclosporine and Tacrolimus in Rats. Adv. Clin. Exp. Med..

[37] Knops N., Van den Heuvel L.P., Masereeuw R., Bongaers I., De Loor H., Levtchenko E., Kuypers D. (2015). The functional implications of common genetic variation in CYP3A5 and ABCB1 in human proximal tubule cells. Mol. Pharm..

[38] Elens L., Capron A., Kerckhove V.V., Lerut J., Mourad M., Lison D., Wallemacq P., Haufroid V. (2007). 1199G>A and 2677G>T/A polymorphisms of ABCB1 independently affect tacrolimus concentration in hepatic tissue after liver transplantation. Pharm. Genom..

[39] Iwasaki K. (2007). Metabolism of tacrolimus (FK506) and recent topics in clinical pharmacokinetics. Drug Metab. Pharm..

[40] Vincent S.H., Karanam B.V., Painter S.K., Chiu S.H. (1992). In vitro metabolism of FK-506 in rat, rabbit, and human liver microsomes: Identification of a major metabolite and of cytochrome P450 3A as the major enzymes responsible for its metabolism. Arch. Biochem. Biophys..

[41] Chen Y.L., Hirabayashi H., Akhtar S., Pelzer M., Kobayashi M. (2006). Simultaneous determination of three isomeric metabolites of tacrolimus (FK506) in human whole blood and plasma using high performance liquid chromatography-tandem mass spectrometry. J. Chromatogr. B Anal. Technol. Biomed. Life Sci..

[42] Dubbelboer I.R., Pohanka A., Said R., Rosenborg S., Beck O. (2012). Quantification of tacrolimus and three demethylated metabolites in human whole blood using LC-ESI-MS/MS. Drug Monit..

[43] Andrews L.M., Li Y., De Winter B.C.M., Shi Y.Y., Baan C.C., Van Gelder T., Hesselink D.A. (2017). Pharmacokinetic considerations related to therapeutic drug monitoring of tacrolimus in kidney transplant patients. Expert Opin. Drug Metab. Toxicol..

[44] Tajima S., Fu R., Shigematsu T., Noguchi H., Kaku K., Tsuchimoto A., Okabe Y., Masuda S. (2019). Urinary Human Epididymis Secretory Protein 4 as a Useful Biomarker for Subclinical Acute Rejection Three Months after Kidney Transplantation. Int. J. Mol. Sci..

